# Impacts of acquisition and reconstruction parameters on the absolute technetium quantification of the cadmium–zinc–telluride-based SPECT/CT system: a phantom study

**DOI:** 10.1186/s40658-021-00412-4

**Published:** 2021-09-26

**Authors:** Ruyi Zhang, Miao Wang, Yaqian Zhou, Shen Wang, Yiming Shen, Ning Li, Peng Wang, Jian Tan, Zhaowei Meng, Qiang Jia

**Affiliations:** Department of Nuclear Medicine, Tianjin Medical University General Hospital, Anshan Road No. 154, Heping District, Tianjin, 300052 People’s Republic of China

**Keywords:** CZT SPECT/CT, Absolute quantification, Recovery coefficient, Full width at half maximum, Attenuation correction, Scatter correction, Resolution recovery correction, OSEM

## Abstract

**Background:**

The digital cadmium–zinc–telluride (CZT)-based SPECT system has many advantages, including better spatial and energy resolution. However, the impacts of different acquisition and reconstruction parameters on CZT SPECT quantification might still need to be validated. This study aimed to evaluate the impacts of acquisition parameters (the main energy window and acquisition time per frame) and reconstruction parameters (the number of iterations, subsets in iterative reconstruction, post-filter, and image correction methods) on the technetium quantification of CZT SPECT/CT.

**Methods:**

A phantom (PET NEMA/IEC image quality, USA) was filled with four target-to-background (T/B) ratios (32:1, 16:1, 8:1, and 4:1) of technetium. Mean uptake values (the calculated mean concentrations for spheres) were measured to evaluate the recovery coefficient (RC) changes under different acquisition and reconstruction parameters. The corresponding standard deviations of mean uptake values were also measured to evaluate the quantification error. Image quality was evaluated using the National Electrical Manufacturers Association (NEMA) NU 2–2012 standard.

**Results:**

For all T/B ratios, significant correlations were found between iterations and RCs (*r* = 0.62–0.96 for 1–35 iterations, *r* = 0.94–0.99 for 35–90 iterations) as well as between the full width at half maximum (FWHM) of the Gaussian filter and RCs (*r* = − 0.86 to − 1.00, all *P* values < 0.05). The regression coefficients of 1–35 iterations were higher than those of 35–90 iterations (0.51–1.60 vs. 0.02–0.19). RCs calculated with AC (attenuation correction) + SC (scatter correction) + RR (resolution recovery correction) combination were more accurate (53.82–106.70%) than those calculated with other combinations (all *P* values < 0.05). No significant statistical differences (all *P* values > 0.05) were found between the 15% and 20% energy windows except for the 32:1 T/B ratio (*P* value = 0.023) or between the 10 s/frame and 120 s/frame acquisition times except for the 4:1 T/B ratio (*P* value = 0.015) in terms of RCs.

**Conclusions:**

CZT-SPECT/CT of technetium resulted in good quantification accuracy. The favourable acquisition parameters might be a 15% energy window and 40 s/frame of acquisition time. The favourable reconstruction parameters might be 35 iterations, 20 subsets, the AC + SC + RR correction combination, and no filter.

**Supplementary Information:**

The online version contains supplementary material available at 10.1186/s40658-021-00412-4.

## Background

Single-photon emission computed tomography (SPECT) has been widely used to diagnose various kinds of human diseases, such as myocardial diseases, endocrine disorders, and central nervous system diseases, since its invention in the 1990s [1–4]. Most of the available SPECT systems are based on the well-known Anger camera with NaI (Tl) as a scintillation material, which determines the position of an event by the centroid of the scintillation light [5]. NaI (Tl)-based detectors capture γ photons and convert the photons into electrons, which are further amplified into strong electrical signals via photomultiplier tubes (PMTs). This conversion process introduces errors, including photon loss, motion artefacts of long acquisition time and higher radiation dosages. In recent years, digital radiographic imaging has certainly replaced analogue imaging. Digital imaging has many advantages, such as better image contrast and image enhancement [6]. In contrast to NaI SPECT, a novel digital cadmium–zinc–telluride (CZT)-based SPECT equipped with solid-state detectors generates electrical signals directly by turning incident γ photons into electron–hole pairs under a high-voltage electric field [7]. This process avoids photon loss and produces better image quality due to its higher spatial and energy resolutions compared to those of NaI SPECT [8]. CZT SPECT also provides a shorter acquisition time and a lower radiation dosage [9, 10].

Absolute quantification was originally applied in positron emission computed tomography (PET). It is considered the gold standard of non-invasive quantification analysis methods for some diseases, such as coronary artery disease, microvascular disease and tumours, due to its high quantification accuracy [11, 12]. PET images display the distribution of certain radionuclides in three dimensions (3D). The data used for PET image reconstructions are in units of radioactivity per unit volume (kBq·cm^−3^), and these data are close to the actual in vivo distribution of the radionuclide. Both PET and SPECT quantification are compromised by three major confounding variables: photon scatter, photon attenuation, and partial volume effect [13]. Scattered photons might fall into the energy window of the PET or SPECT system and therefore affect the overall quantification and image quality [14, 15]. Attenuation may lead to artefacts and inaccuracies in reconstructed images due to the highly non-uniform distribution of attenuating tissues [16]. Partial-volume effects can lead to spillover between two adjacent regions, generally resulting in the tracer uptake being underestimated. Smaller lesions often suffer severely from this effect [17, 18]. These problems were solved and validated in PET several decades ago because of the advantages offered by positron decay and coincidence detection [19, 20]. These problems have also subsequently been solved to some extent in SPECT. Several methods have been applied in scatter correction (SC), including the deconvolution method [21, 22], energy window subtraction method [23, 24], energy-weighted acquisition method [25, 26], inverse Monte Carlo reconstruction algorithm, and so on [27, 28]. Attenuation correction (AC) has been achieved by using the CT-based attenuation correction method [29], Chang algorithm method [30], and so on. Additionally, some methods have been applied to reduce the partial volume effect, including image enhancement techniques [31, 32], image domain anatomically-based PVC (partial volume correction) techniques [33], projection-based PVC, and so on [34]*.* Despite this, both the spatial and energy resolutions of conventional NaI SPECT are relatively low, and radionuclides applied in SPECT have a higher fraction of scattered photons than those of PET [35, 36]. These drawbacks might magnify the partial volume effect and compromise the effectiveness of SC and AC. As a result, SPECT images may be more difficult to quantify.

Today, however, with the development of SPECT systems, absolute quantification is also widely validated and used. Some studies have suggested that absolute SPECT quantification is promising with different SPECT equipment when reconstruction protocols are standardized [37, 38]. SPECT quantification of various radionuclides has also been well studied, including technetium-99 m (^99m^Tc) [39], indium-111 (^111^In) [40], yttrium-90 (^90^Y) [41], lutetium-177 (^177^Lu) and so on [42]. Additionally, SPECT quantification has been widely used in clinical practice, such as quantification of the lung shunt fraction in hepatic radioembolization [43], myocardial perfusion imaging [44], monitoring cancer [45], and determining lesion volumes [46]. Despite these validations and clinical practices, various acquisition and reconstruction parameters may also affect the accuracy of SPECT quantification. Some studies have suggested that the small number of iterations and subsets used in OSEM (ordered subsets expectation maximization) reconstruction influences the quantification accuracy because of incomplete convergence [47, 48]. The application of different correction methods, such as AC, SC, and RR (resolution recovery correction), may also affect the quantification accuracy [29, 49, 50]. However, many of these studies are based on conventional NaI SPECT systems, and therefore, the impacts of different acquisition and reconstruction parameters on absolute CZT SPECT quantification might need to be studied. This study aimed to evaluate the impacts of acquisition parameters (the main acquisition energy window and acquisition time/frame) and reconstruction parameters (the number of iterations and subsets in iterative reconstruction, post-filter, AC, SC, and RR correction) on the accuracy of CZT SPECT/CT technetium quantification.

## Materials and methods

### Phantom preparation

The phantom (NEMA/IEC 2001) used for this experiment consisted of a D-shaped cylinder and six spheres with different diameters (37 mm, 28 mm, 22 mm, 17 mm, 13 mm, and 10 mm, Additional file 1: Fig. S1). We filled the phantom with ^99m^TcO_4_^−^ (Atomic Technology Corporation, China) at four target-to-background (T/B) ratios (32:1, 16:1, 8:1, and 4:1). The radioactivity in spheres of four T/B ratios was 0.20 MBq/ml (32:1), 0.11 MBq/ml (16:1), 0.06 MBq/ml (8:1), and 0.03 MBq/ml (4:1), respectively, by the time of acquisition. The decay of ^99m^TcO_4_^−^ was calibrated to the time of acquisition [51].

### Image acquisition parameters

SPECT/CT acquisition of the PET NEMA/IEC image quality phantom was performed on a Discovery NM/CT 670 CZT (GE Healthcare, USA) equipped with wide energy high-resolution collimators. All SPECT images were acquired with a list mode. The step and shoot acquisition mode was performed by 360-degree rotations (120 s/6-degree per frame) with a matrix size of 128 × 128 without zoom. Two main energy windows (140 keV ± 7.5% and 140 keV ± 10%) were reconstructed by the list mode to evaluate the impacts of the main energy window on RCs. The scatter energy window was 120 ± 5% keV. CT images were acquired with a 120 kVp tube voltage, 200 mA tube current, matrix size of 512 × 512, and 1.25 mm slice thickness.

### Image reconstruction parameters

**Fig. 1:**
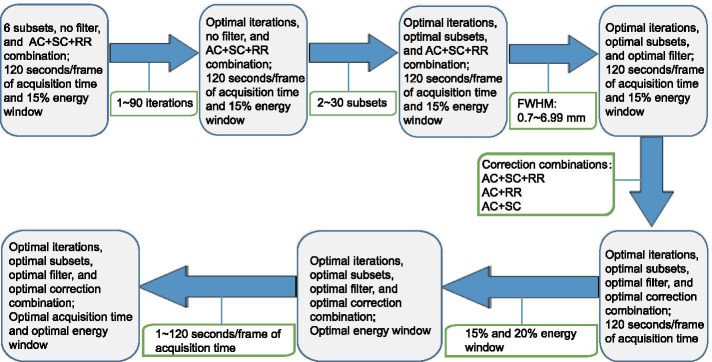
Analysis process of different acquisition and reconstruction parameters. The process started with six subsets, no filter, AC + SC + RR correction combination, 120 s/frame of acquisition time, and 15% energy window. The impacts of iterations, subsets, FWHM, correction combination, energy window, and acquisition time/frame were evaluated in sequence and an optimal value of these parameters was determined in each step of the process

All images were reconstructed using the OSEM algorithm with 1–90 iterations and 2–30 subsets [52]. The FWHM range of the Gaussian filter was 0.7–6.99 mm. The correction methods used in this study included CT-based AC, dual-energy-window technique-based SC, and point spread function-based RR correction. Three image correction combinations were used to evaluate the impacts of the image correction methods, including AC + SC + RR, AC + SC, and AC + RR. List mode was applied to reconstruct the acquisition time to 1–120 s/frame. In every step of the analysis, we evaluated the impact of a certain parameter to determine the optimal value while fixing all other parameters at the same time. All acquisition and reconstruction parameters in the evaluation process are listed in Fig. 1.

## Quantitative analysis

### RC calculations

Volumes of interest (VOIs) of six spheres were delineated using the inner edge of spheres of CT images as references. Mean uptake values (MBq/ml) and corresponding standard deviations (SDs) were automatically calculated three times by the Q. Metrix of GE-Xeleris 4.0 workstation (GE Healthcare, USA) and are shown as averages. RCs were calculated using Eq. (1) [19]:

1 $$RC=\frac{\mathrm{Mean\,measured\,radioactivity\,concentration}}{\mathrm{Actual\,radioactivity\,concentration}}\times 100\%.$$

### Image quality evaluations

To assess the image quality, we calculated both the per cent contrast and coefficient of variation (COV) complying with the NEMA NU 2–2012 standard [53–56]. The per cent contrast *Q*_*H,j*_ for each hot sphere was calculated by using Eq. (2):

2 $${Q}_{\mathrm{H},j}=\frac{ {C}_{\mathrm{H},j}/{C}_{\mathrm{B},j}-1}{{a}_{\mathrm{H}}/{a}_{\mathrm{B}}-1}\times 100\%.$$

where *C*_H*,j*_ is the average count in the region of interest (ROI) for sphere *j*, *C*_B*,j*_ is the average of the background ROI counts for sphere *j*, *a*_H_ is the activity concentration in the hot spheres, and *a*_B_ is the activity concentration in the background.

The COV *N*_*j*_ for each hot sphere was calculated by using Eq. (3):

3 $${N}_{j}=\frac{ {\mathrm{SD}}_{j}}{{C}_{\mathrm{B},j}}\times 100\%.$$

where SD_*j*_ is the standard deviation of the background ROI counts for sphere *j*.

### Statistical analysis

All statistical analyses were performed by SPSS 23.0 (IBM, USA). All graphs were produced by GraphPad Prism 8.3.0 (GraphPad Software, USA) and Origin Pro 2021 (OriginLab, USA). The relationships between RCs and the different number of iterations and subsets, FWHM, and acquisition time/frame were established by Pearson’s rank correlation and linear regression analysis. RCs and per cent contrast of three different correction combinations were compared using the paired t-test [57]. The comparison of RCs for different energy windows and acquisition time/frame was also analysed by using the paired t-test. A *P* value lower than 0.05 was considered statistically significant.

## Results

### Impacts of the number of iterations and subsets

**Fig. 2:**
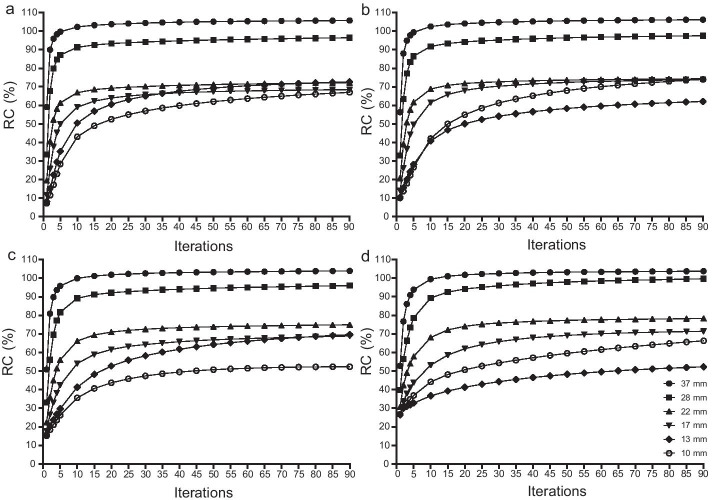
Impacts of iterations on RCs. **a** 32:1 T/B ratio; **b** 16:1 T/B ratio; **c** 8:1 T/B ratio; **d** 4:1 T/B ratio; RCs were calculated with 1–90 iterations; Fixed reconstruction parameters: six subsets, no filter, AC + SC + RR combination; Fixed acquisition parameters: 120 s/frame of acquisition time, 15% energy window

Figure 2 shows that of all the T/B ratios, the RCs of larger spheres converged earlier than those of smaller spheres, of which 37–17 mm spheres converged at 35 iterations and 13–10 mm spheres converged at 85 iterations. Table 1 shows that apart from the 37 mm sphere of 1–35 iterations of the 32:1 and 16:1 T/B ratios, there were significant positive correlations between RCs and iterations (*r* = 0.62–0.96 for 1–35 iterations, *r* = 0.94–0.99 for 35–90 iterations, all *P* values < 0.05). Linear regression analysis shows that 1–35 iterations had much higher regression coefficients than those of 35–90 iterations (0.63–1.60 vs. 0.02–0.15 for the 32:1 T/B ratio, 0.70–1.59 vs. 0.02–0.15 for the 16:1 T/B ratio, 0.80–1.29 vs. 0.02–0.17 for the 8:1 T/B ratio, and 0.51–1.26 vs. 0.02–0.19 for the 4:1 T/B ratio). RCs increased rapidly within the first 35 iterations.

**Table 1:** Correlation and linear regression analysis of RCs among different acquisition and reconstruction parameters

			37 mm	28 mm	22 mm	17 mm	13 mm	10 mm
32:1	Iterations (1–35)	Regression coefficient	0.63	0.93	0.93	1.21	1.60	1.47
		*r*	0.58	0.62*	0.70*	0.80*	0.90**	0.92**
	Iterations (35–90)	Regression coefficient	0.02	0.03	0.03	0.04	0.11	0.15
		*r*	0.98**	0.99**	0.99**	0.97**	0.97**	0.99**
	Subsets	Regression coefficient	0.09	0.15	0.20	0.39	0.69	0.70
		*r*	0.68	0.80*	0.87*	0.89*	0.84*	0.85*
	FWHM	Regression coefficient	− 11.36	− 11.83	− 9.96	− 10.28	− 10.88	− 9.49
		*r*	− 1.00**	− 0.99**	− 0.98**	− 0.96**	− 0.92*	− 0.87*
	Acquisition time (s/frame)	Regression coefficient	− 0.01	0.04	0.07	0.05	0.05	0.05
		*r*	− 0.62	0.84*	0.72*	0.66	0.36	0.36
16:1	Iterations (1–35)	Regression coefficient	0.70	1.04	1.03	1.37	1.28	1.59
		*r*	0.59	0.66*	0.74*	0.84*	0.92**	0.95**
	Iterations (35–90)	Regression coefficient	0.02	0.03	0.03	0.04	0.11	0.15
		*r*	0.98**	0.98**	0.98**	0.98**	0.98**	0.98**
	Subsets	Regression coefficient	0.07	0.12	0.13	0.35	0.69	1.14
		*r*	0.61	0.64	0.70	0.76*	0.86*	0.90*
	FWHM	Regression coefficient	− 11.02	− 11.83	− 9.85	− 10.65	− 8.68	− 10.09
		*r*	− 1.00**	− 0.99**	− 0.99**	− 0.96**	− 0.92*	− 0.86*
	Acquisition time (s/frame)	Regression coefficient	− 0.04	− 0.03	− 0.02	− 0.04	0.02	0.28
		*r*	− 0.63	− 0.55	− 0.73*	− 0.68	0.29	0.82*
8:1	Iterations (1–35)	Regression coefficient	0.80	1.15	1.15	1.23	1.29	0.98
		*r*	0.63*	0.72*	0.82*	0.88**	0.95**	0.94**
	Iterations (35–90)	Regression coefficient	0.02	0.04	0.03	0.06	0.17	0.06
		*r*	0.98**	0.99**	0.98**	0.98**	0.98**	0.94**
	Subsets	Regression coefficient	0.08	0.10	0.26	0.47	0.90	0.40
		*r*	0.65	0.52	0.83*	0.85*	0.83*	0.66
	FWHM	Regression coefficient	− 9.95	− 10.90	− 8.73	− 8.79	− 9.22	− 6.23
		*r*	− 1.00**	− 0.99**	− 0.98**	− 0.96**	− 0.92*	− 0.90*
	Acquisition time (s/frame)	Regression coefficient	0.09	0.04	− 0.02	0.01	0.12	− 0.31
		*r*	0.70	0.62	− 0.44	0.02	0.43	− 0.71*
4:1	Iterations (1–35)	Regression coefficient	0.87	1.26	1.10	1.09	0.51	0.81
		*r*	0.69*	0.81*	0.85**	0.92**	0.96**	0.95**
	Iterations (35–90)	Regression coefficient	0.02	0.05	0.03	0.07	0.12	0.19
		*r*	0.98**	0.98**	0.97**	0.96**	0.99**	0.99**
	Subsets	Regression coefficient	− 0.06	0.11	0.16	0.50	0.56	1.14
		*r*	− 0.34	0.44	0.57	0.76*	0.87*	0.98**
	FWHM	Regression coefficient	− 8.59	− 9.39	− 7.24	− 7.51	− 4.23	− 6.10
		*r*	− 1.00**	− 0.99**	− 0.99**	− 0.96**	− 0.90*	− 0.89*
	Acquisition time (s/frame)	Regression coefficient	0.16	0.21	− 0.08	0.24	0.26	− 0.34
		*r*	0.71*	0.76*	− 0.68	0.82*	0.70	− 0.53

**, *P*-value < 0.001; *, *P*-value < 0.05; *r*, Pearson *r*

**Fig. 3:**
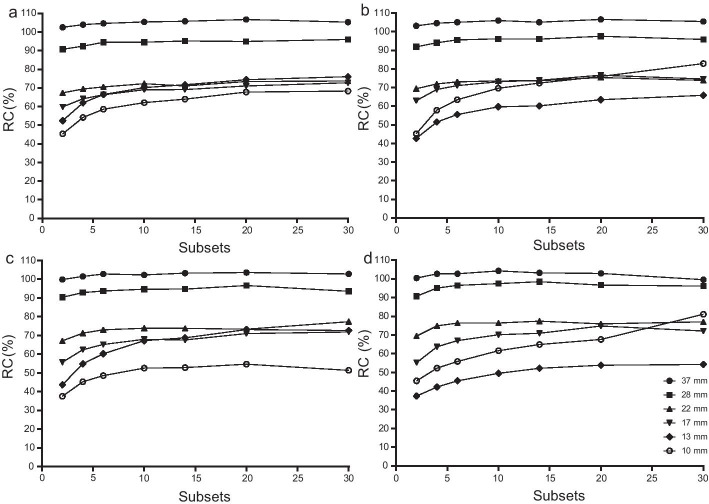
Impacts of subsets on RCs. **a** 32:1 T/B ratio; **b** 16:1 T/B ratio; **c** 8:1 T/B ratio; **d** 4:1 T/B ratio; RCs were calculated with 2–30 subsets; Fixed reconstruction parameters: 35 iterations, no filter, AC + SC + RR combination; Fixed acquisition parameters: 120 s/frame of acquisition time, 15% energy window

Figure 3 shows that RCs did not increase rapidly with an increasing number of subsets. RCs of larger spheres (37–17 mm) became stable after 20 subsets. Table 1 shows that the Pearson *r* values of the six spheres ranged from 0.68 to 0.89 (32:1 T/B ratio), 0.61 to 0.90 (16:1 T/B ratio), 0.52 to 0.85 (8:1 T/B ratio), and − 0.34 to 0.98 (4:1 T/B ratio). In linear regression analysis, the regression coefficients of the six spheres ranged from 0.09 to 0.70 for the 32:1 T/B ratio, 0.07 to 1.14 for the 16:1 T/B ratio, 0.08 to 0.90 for the 8:1 T/B ratio, and − 0.06 to 1.14 for the 4:1 T/B ratio.

### Impacts of the Gaussian filter

**Fig. 4:**
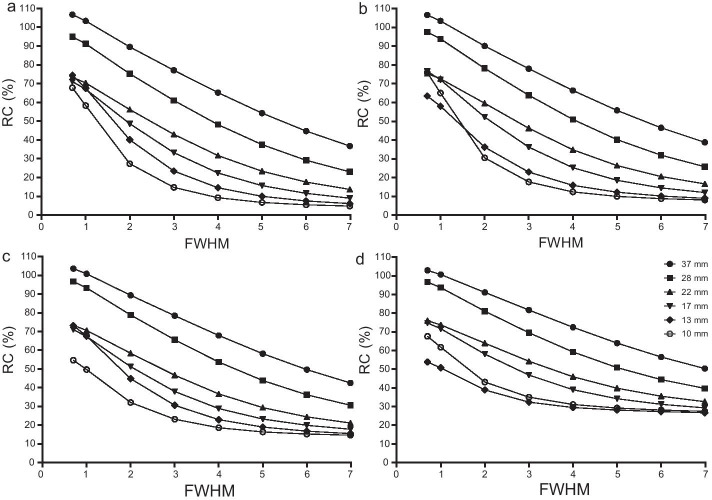
Impacts of FWHM value of the Gaussian filter on RCs. **a** 32:1 T/B ratio; **b** 16:1 T/B ratio; **c** 8:1 T/B ratio; **d** 4:1 T/B ratio; RCs were calculated with FWHM value of 0.70–6.99; Fixed reconstruction parameters: 35 iterations, 20 subsets, AC + SC + RR combination; Fixed acquisition parameters: 120 s/frame of acquisition time, 15% energy window

In Fig. 4, RCs of all spheres declined significantly as the FWHM (0.7–6.99 mm) of the Gaussian filter increased. There were significant negative correlations between the FWHM of the Gaussian filter and RCs for all spheres (*r*: − 0.87 to − 1.00 for the 32:1 T/B ratio, − 0.86 to  − 1.00 for the 16:1 T/B ratio, − 0.90 to  − 1.00 for the 8:1 T/B ratio, and − 0.89 to  − 1.00 for the 4:1 T/B ratio, all *P* values < 0.05). Additionally, there were high regression coefficients in all 6 spheres (− 9.49 to  − 11.83 for the 32:1 T/B ratio, − 8.68 to  − 11.83 for the 16:1 T/B ratio, − 6.23 to  − 10.90 for the 8:1 T/B ratio, and − 4.23 to  − 9.39 for the 4:1 T/B ratio, Table 1). Furthermore, there was no FWHM plateau in terms of RCs, which was different from iterations or subsets.

### Impacts of the image correction methods

**Fig. 5:**
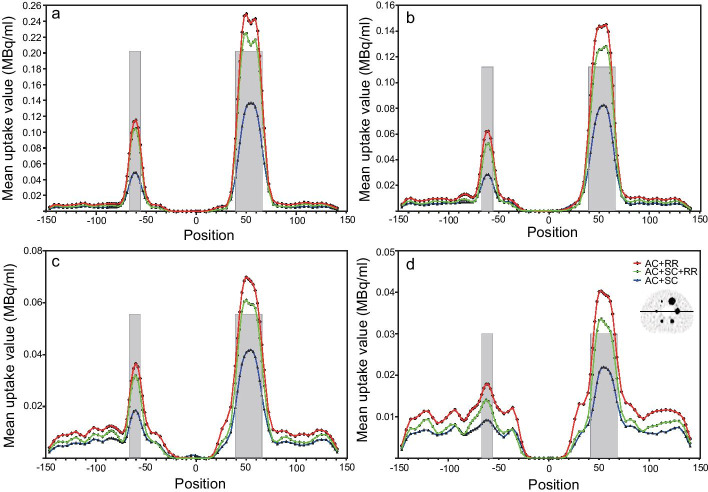
Location of line profiles of 32:1 (**a**), 16:1 (**b**), 8:1 (**c**), and 4:1 (**d**) T/B ratios through the central slice of the entire phantom. X-axis indicates the pixel position of the phantom; Y-axis indicates the calculated mean uptake values of the phantom; The upper edges of both grey areas are the actual radioactivity concentration in spheres; Different curves were calculated mean uptake values by using different correction combinations (AC + SC + RR, AC + SC, and AC + RR); Fixed reconstruction parameters: 35 iterations, 20 subsets, no filter; Fixed acquisition parameters: 120 s/frame of acquisition time, 15% energy window

**Fig. 6:**
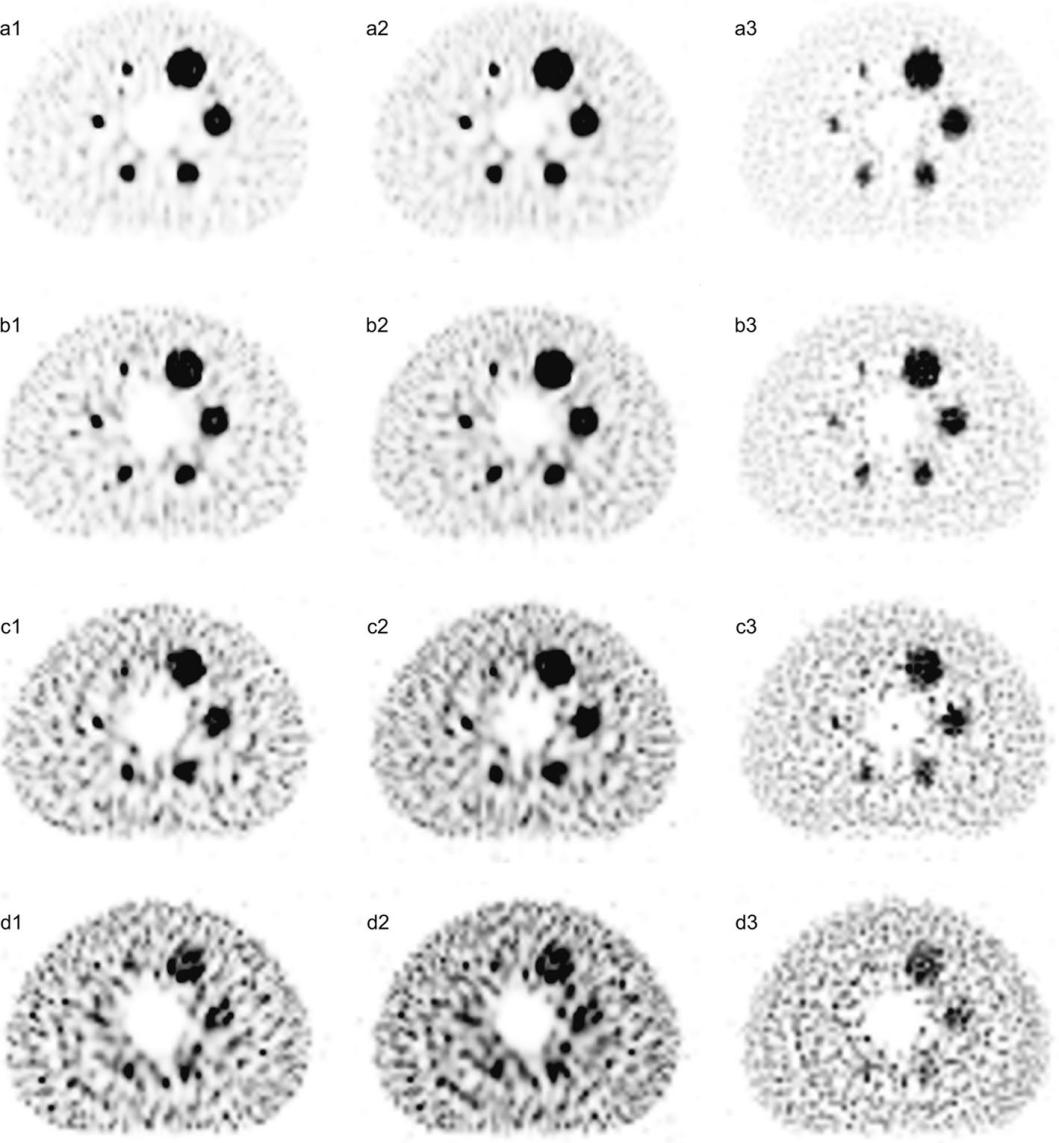
Images reconstructed with different image correction combinations. Rows were the images of different T/B ratios (32:1, 16:1, 8:1, and 4:1, respectively, from the top to the bottom; **a** 32:1; **b** 16:1; **c** 8:1; **d** 4:1); Columns are the images reconstructed with different correction combinations (1, AC + SC + RR; 2, AC + RR; 3, AC + SC). Fixed reconstruction parameters: 35 iterations, 20 subsets, no filter; Fixed acquisition parameters: 120 s/frame of acquisition time, 15% energy window

The profiles of four T/B ratios show that RCs of the AC + SC + RR correction combination were closer to the actual sphere activity concentration. The AC + RR combination predicted the highest mean uptake values, while the AC + SC combination predicted the lowest mean uptake values in spheres (Fig. 5). Table 2 shows that the RCs of the AC + SC + RR combination were lower than those of the AC + RR combination but higher than those of the AC + SC combination (67.80–106.70% vs. 75.68–120.23% vs. 29.91–67.96% for the 32:1 T/B ratio; 63.44–106.57% vs. 72.80–122.00% vs. 30.89–71.98% for the 16:1 T/B ratio; 54.67–103.58% vs. 62.93–120.84% vs. 28.21–71.91% for the 8:1 T/B ratio; 53.82–102.89% vs. 66.64–123.41% vs. 34.34–73.86% for the 4:1 T/B ratio, all *P* values < 0.05). Figure 6 shows the visual difference of the images reconstructed with different correction combinations. Among all T/B ratios, the AC + SC + RR combination had a better visual image quality. Table 3 shows that the per cent contrasts of six spheres reconstructed using the AC + SC + RR combination were higher than those of other correction combinations (AC + SC + RR vs. AC + RR; AC + SC + RR vs. AC + SC, all *P* values < 0.05). However, the COVs of the AC + RR combination were lower than those of the AC + SC + RR combination or AC + RR combination (all *P* values < 0.05). The COVs of the AC + SC combination were higher than those of the AC + SC + RR combination (100.70–103.52% vs. 85.95–93.77% for the 32:1 T/B ratio, 94.43–97.64% vs. 85.71–95.09% for the 16:1 T/B ratio, 93.75–96.31% vs. 87.10–97.25% for the 8:1 T/B ratio, 91.38–94.04% vs. 79.55–92.71% for the 4:1 T/B ratio, *P* values of 32:1, 16:1, and 4:1 T/B ratios < 0.05, Table 4).

**Table 2:** Comparison of RCs (%) of all spheres among three different correction combinations

	Correction combination	37 mm	28 mm	22 mm	17 mm	13 mm	10 mm	*P* value
32:1	AC + SC + RR	106.70	94.94	73.44	71.06	74.39	67.80	
	AC + RR	120.23	107.38	82.77	78.93	80.52	75.68	< 0.001^a^
	AC + SC	67.96	53.86	41.65	34.67	29.91	31.02	< 0.001^b^
16:1	AC + SC + RR	106.57	97.53	75.37	76.64	63.44	75.91	
	AC + RR	122.00	109.81	84.78	87.25	72.80	81.33	< 0.001^a^
	AC + SC	71.98	59.22	43.23	41.91	30.89	32.54	< 0.001^b^
8:1	AC + SC + RR	103.58	96.65	73.31	70.98	73.14	54.67	
	AC + RR	120.84	112.18	88.17	84.09	81.65	62.93	< 0.001^a^
	AC + SC	71.91	60.81	45.30	41.41	38.88	28.21	< 0.001^b^
4:1	AC + SC + RR	102.89	96.64	75.93	74.79	53.82	67.59	
	AC + RR	123.41	115.88	92.70	88.94	66.64	75.21	< 0.001^a^
	AC + SC	73.86	62.46	51.10	44.19	34.34	39.69	< 0.001^b^

Reconstruction parameters: 35 iterations, 20 subsets, no filter; Acquisition parameters: 120 s/frame of acquisition time, 15% main energy window; AC, attenuation correction; SC, scatter correction; RR, resolution recovery correction; a, AC + SC + RR vs. AC + RR; b, AC + SC + RR vs. AC + SC

**Table 3:** Comparison of per cent contrasts (%) of all spheres among three different correction combinations

	Correction combination	37 mm	28 mm	22 mm	17 mm	13 mm	10 mm	*P* value
32:1	AC + SC + RR	88.95	80.36	77.35	71.82	59.65	54.13	
	AC + RR	73.37	65.86	64.64	56.71	46.45	43.12	< 0.001^a^
	AC + SC	58.30	42.81	32.41	15.69	8.91	7.41	< 0.001^b^
16:1	AC + SC + RR	92.82	79.25	85.58	62.21	59.91	48.74	
	AC + RR	76.85	64.98	71.41	51.78	51.15	37.85	< 0.001^a^
	AC + SC	80.79	66.69	60.76	43.97	28.20	23.83	0.001^b^
8:1	AC + SC + RR	78.65	71.05	64.37	57.63	43.49	39.34	
	AC + RR	69.73	65.77	50.55	52.58	37.50	32.00	0.002^a^
	AC + SC	77.93	63.92	38.94	38.93	28.40	22.11	0.011^b^
4:1	AC + SC + RR	99.94	76.67	72.83	66.09	45.03	65.30	
	AC + RR	80.81	62.67	64.85	51.79	39.67	45.96	0.002^a^
	AC + SC	77.03	63.33	41.89	38.29	9.28	40.53	< 0.001^b^

Reconstruction parameters: 35 iterations, 20 subsets, no filter; Acquisition parameters: 120 s/frame of acquisition time, 15% energy window; AC, attenuation correction; SC, scatter correction; RR, resolution recovery correction; a, AC + SC + RR vs. AC + RR; b, AC + SC + RR vs. AC + SC

**Table 4:** Comparison of COVs (Coefficients of variation, %) of all spheres among three different correction combinations

	Correction combination	37 mm	28 mm	22 mm	17 mm	13 mm	10 mm	*P* value
32:1	AC + SC + RR	93.77	92.21	88.01	87.85	85.95	86.64	
	AC + RR	74.53	72.91	70.50	71.00	69.76	70.82	< 0.001^a^
	AC + SC	102.96	103.52	101.71	101.53	100.70	100.80	< 0.001^b^
16:1	AC + SC + RR	95.09	94.61	89.02	89.62	88.86	85.71	
	AC + RR	76.36	75.60	72.28	72.54	70.40	69.06	< 0.001^a^
	AC + SC	96.95	96.33	97.64	97.13	95.44	94.43	0.007^b^
8:1	AC + SC + RR	97.25	94.93	91.36	90.93	90.74	87.10	
	AC + RR	77.98	75.92	73.78	72.54	72.06	69.67	< 0.001^a^
	AC + SC	96.31	93.75	94.79	94.89	95.34	95.60	0.096^b^
4:1	AC + SC + RR	92.71	88.97	84.03	83.13	82.02	79.55	
	AC + RR	73.12	72.69	69.53	68.38	66.97	64.92	< 0.001^a^
	AC + SC	91.77	91.38	91.91	91.49	91.80	94.04	0.026^b^

Reconstruction parameters: 35 iterations, 20 subsets, no filter; Acquisition parameters: 120 s/frame of acquisition time, 15% energy window; AC, attenuation correction; SC, scatter correction; RR, resolution recovery correction; a, AC + SC + RR vs. AC + RR; b, AC + SC + RR vs. AC + SC

### Impacts of the main energy window

As shown in Table 5, for the 32:1 T/B ratio, RCs under the 15% energy window were higher than those under the 20% energy window, and there was a statistically significant difference (*P* value = 0.023). However, for lower T/B ratios (16:1, 8:1, and 4:1), there were no statistically significant differences (all *P* values > 0.05). Since a 15% energy window might improve the quantification accuracy for a higher T/B ratio (32:1), it was determined to be the optimal energy window in this step.

**Table 5:** Comparison of RCs (%) between the 15% energy window and 20% energy window

	Energy window (%)	37 mm	28 mm	22 mm	17 mm	13 mm	10 mm	*P* value
32:1	15	106.70	94.94	73.44	71.06	74.39	67.80	0.023
	20	105.50	94.23	72.93	70.83	72.89	65.26	
16:1	15	106.57	97.53	75.37	76.64	63.44	75.91	0.314
	20	104.94	96.93	75.36	77.38	63.79	74.35	
8:1	15	103.58	96.65	73.31	70.98	73.14	54.67	0.919
	20	102.46	96.73	74.10	74.64	72.02	51.80	
4:1	15	102.89	96.64	75.93	74.79	53.82	67.59	0.933
	20	104.26	96.31	79.16	77.13	57.10	58.69	

Reconstruction parameters: 35 iterations, 20 subsets, No filter; AC + SC + RR correction. Acquisition parameters: 120 s/frame of acquisition time

### Impacts of the acquisition time per frame

**Fig. 7:**
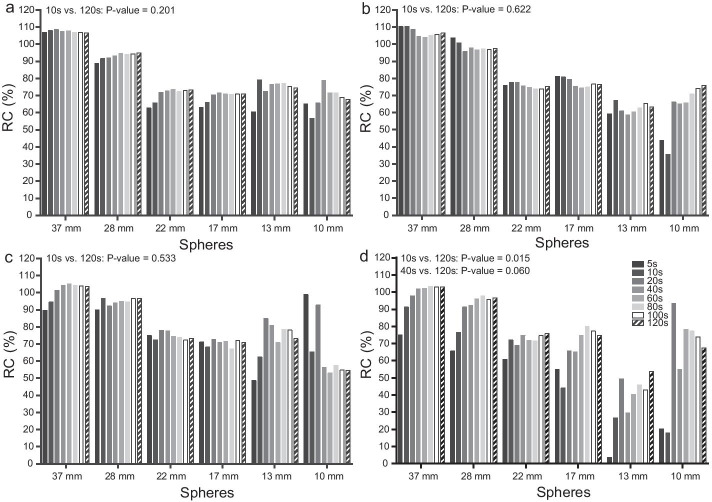
RCs under different acquisition time. **a** 32:1 T/B ratio; **b** 16:1 T/B ratio; **c** 8:1 T/B ratio; **d** 4:1 T/B ratio; X-axis indicates different spheres grouped by size; Y-axis indicates RCs; RCs of each sphere were calculated with 5–120 s/frame of acquisition time (from left to right). *P* values were calculated by using the paired t-test. Fixed reconstruction parameters: 35 iterations, 20 subsets, no filter, AC + SC + RR combination; Fixed acquisition parameters: 15% energy window

**Fig. 8:**
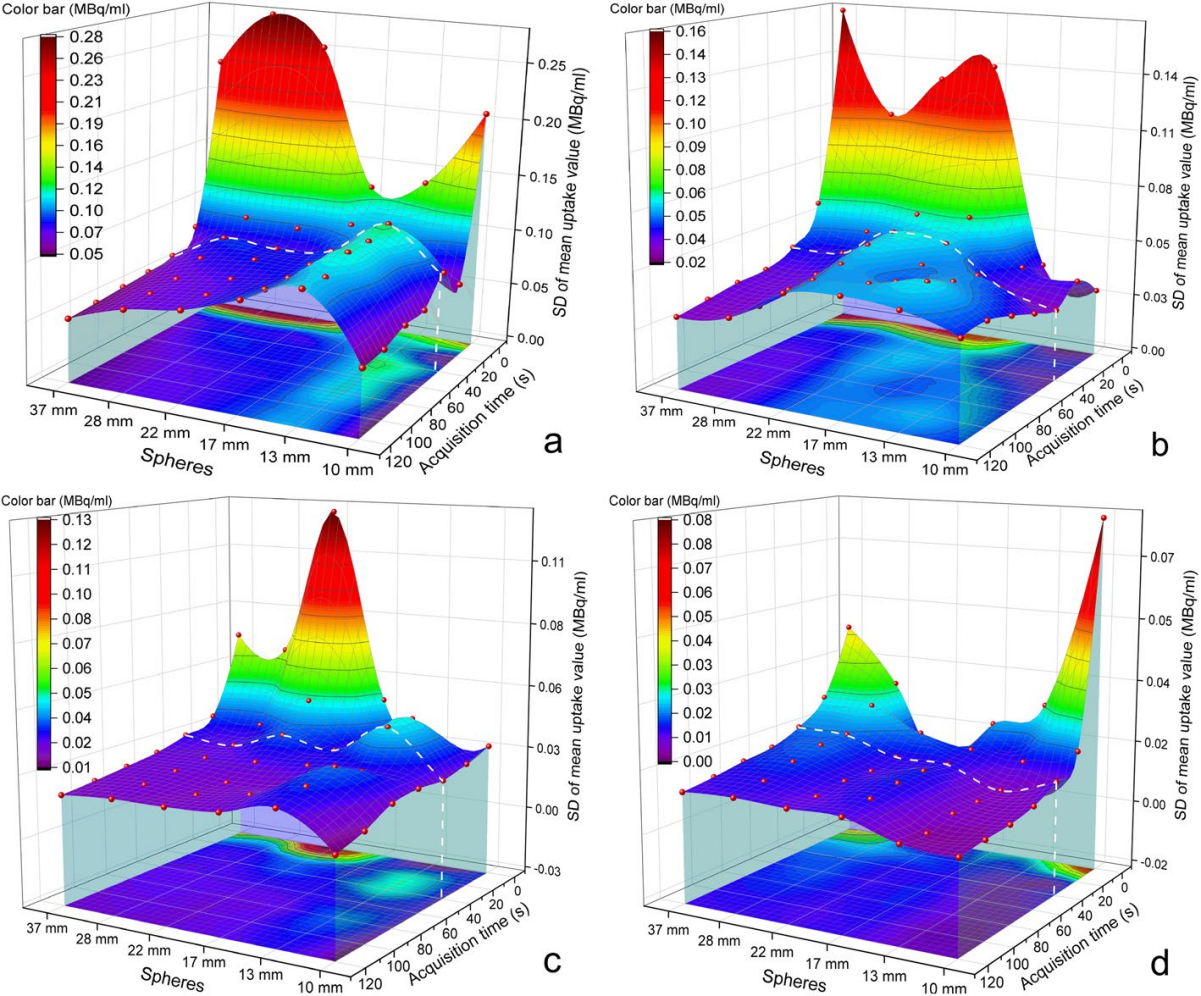
Fitted 3D-scatter plot of standard deviations (SDs) of RCs. **a** 32:1 T/B ratio; **b** 16:1 T/B ratio; **c** 8:1 T/B ratio; **d** 4:1 T/B ratio; X-axis indicates 6 spheres (37 mm, 28 mm, 22 mm, 17 mm, 13 mm, and 10 mm); Y-axis indicates SD of RCs; Z-axis indicates different acquisition time/frame (1–120 s/frame); White dashed line is the proposed cut-off value for acquisition time (40 s/frame). Fixed reconstruction parameters: 35 iterations, 20 subsets, no filter, AC + SC + RR combination; Fixed acquisition parameters: 15% energy window

The correlations between RCs and the acquisition time/frame were − 0.62 to 0.84 for the 32:1 T/B ratio, − 0.73 to 0.82 for the 16:1 T/B ratio, − 0.71 to 0.70 for the 8:1 T/B ratio, and − 0.68 to 0.82 for the 4:1 T/B ratio. Regression coefficients between RCs and the acquisition time/frame were − 0.01 to 0.07 for the 32:1 T/B ratio, − 0.04 to 0.28 for the 16:1 T/B ratio, − 0.31 to 0.12 for the 8:1 T/B ratio, and − 0.34 to 0.26 for the 4:1 T/B ratio (Table 1). Figure 7 shows that for 32:1, 16:1 and 8:1 T/B ratios, RCs did not increase significantly with increasing acquisition time/frame (all *P* values > 0.05 when comparing 10 s with 120 s/frame of acquisition time). However, for the 4:1 T/B ratio, the RCs of six spheres increased significantly within the first 40 s/frame of acquisition time (10 s vs. 120 s, *P* value = 0.015; 40 s vs. 120 s, *P* value = 0.060). Figure 8 shows that for most spheres of all T/B ratios, the SD of the mean uptake values decreased significantly in the first 40 s/frame of acquisition time and then remained stable. Table 6 shows that the RCs were 71.78–107.65% for the 32:1 T/B ratio, 58.92–104.55% for the 16:1 T/B ratio, 56.57–104.44% for the 8:1 T/B ratio, 29.80–102.02% for the 4:1 T/B ratio under the optimal 40 s/frame of acquisition time, and other optimal reconstruction and acquisition parameters as follows: 35 iterations, 20 subsets, no filter, AC + SC + RR combination and 15% energy window.

**Table 6:** RCs (%) of different acquisition time

	Time (s/frame)	37 mm	28 mm	22 mm	17 mm	13 mm	10 mm
32:1	5	106.95	88.73	62.68	63.13	60.54	65.16
	10	107.95	91.69	65.81	65.90	79.24	56.79
	20	108.60	91.89	71.77	70.44	72.52	65.60
	40	107.65	93.33	72.92	71.78	76.66	79.02
	60	107.80	94.66	73.57	70.86	76.84	71.53
	80	107.03	94.16	72.40	70.76	77.06	71.47
	100	106.92	94.40	72.97	70.94	75.35	68.91
	120	106.70	94.94	73.44	71.06	74.39	67.80
16:1	5	110.31	103.77	75.90	81.12	59.25	43.66
	10	110.49	100.87	77.71	80.73	67.05	35.59
	20	108.68	95.65	77.63	79.50	60.86	66.15
	40	104.55	97.85	75.80	75.59	58.92	65.15
	60	103.98	96.69	74.82	74.33	60.26	65.82
	80	105.21	97.14	74.00	75.02	62.69	70.83
	100	105.83	96.80	73.98	76.75	65.52	74.21
	120	106.57	97.53	75.37	76.64	63.44	75.91
8:1	5	89.75	89.79	74.99	71.09	48.65	98.87
	10	94.57	96.78	72.29	68.26	62.37	65.46
	20	101.43	92.18	78.06	72.69	84.84	92.74
	40	104.44	94.10	77.68	70.95	81.08	56.57
	60	105.14	94.96	74.50	71.64	70.88	53.06
	80	104.39	94.64	73.74	67.25	78.64	57.59
	100	103.85	96.58	72.37	72.04	78.16	54.77
	120	103.58	96.65	73.31	70.98	73.14	54.67
4:1	5	75.02	65.68	60.85	54.83	3.72	20.36
	10	91.33	76.63	72.05	44.15	26.79	18.01
	20	97.88	91.30	69.08	65.61	49.50	93.46
	40	102.02	92.37	74.92	65.15	29.80	55.07
	60	102.09	96.05	71.97	74.78	40.34	78.31
	80	103.36	97.70	71.71	79.94	46.03	77.28
	100	103.17	95.81	74.64	77.40	43.13	73.95
	120	102.89	96.64	75.93	74.79	53.82	67.59

Reconstruction parameters: 35 iterations, 20 subsets, No filter; AC + SC + RR correction. Acquisition parameters: 15% energy window

## Discussion

In this study, CT images were applied as references to avoid the partial volume effect of the SPECT system to calculate RCs with lower errors [58]. The study of Dr. Koole, M et al. suggested that high-resolution structural information from MR or CT images is helpful in determining potential lesions in SPECT images [59].

This study showed that the number of iterations had a large impact on quantification. Figure 2 shows that RCs of larger spheres (37–17 mm) converged earlier than those of smaller spheres (13 mm and 10 mm spheres). This indicated that a small number of iterations might be enough for larger lesions in absolute quantification. Although the correlations between RCs and 1–35 iterations were lower than those of 35–90 iterations, 1–35 iterations had much higher regression coefficients than those of 35–90 iterations (Table 1). RCs could also increase rapidly within the first 35 iterations for all spheres (Fig. 2). This indicated that although 35–90 iterations had strong linearity, it could not increase RCs efficiently because of the much smaller regression coefficients compared with those of 1–35 iterations. Furthermore, smaller spheres had relatively larger errors, and more iterations could increase RCs, but not efficiently. Therefore, it was determined that the optimal number of iterations might be 35.

The correlations between subsets and RCs were not obvious because, for many spheres, the *P* values were greater than 0.05 (Table 1). Additionally, the regression coefficients of all T/B ratios were very low (0.09–0.70 for the 32:1 T/B ratio, 0.07–1.14 for the 16:1 T/B ratio, 0.08–0.90 for the 8:1 T/B ratio, and − 0.06 to 1.14 for the 4:1 T/B ratio) (Table 1). These results indicated that RCs could not increase rapidly with the increasing number of subsets; therefore, subsets had a relatively small impact on quantification. The study of Dr. Vriens, D et al. also suggested that subsets have only a small effect on the standardized uptake value (SUV) in phantom experiments [60]. In this study, RCs tended to be stable after 20 subsets for the larger spheres (37–17 mm) and did not increase significantly after 20 subsets for the smaller spheres (13 mm and 10 mm spheres). Therefore, 20 subsets were applied in this study.

Among all reconstruction parameters, the FWHM of the Gaussian filter showed the most significant correlations (all Pearson’s *r* < − 0.85, all *P* values < 0.05) with RCs as well as the highest regression coefficients (− 9.49 to  − 11.83 for the 32:1 T/B ratio, − 8.68 to  − 11.83 for the 16:1 T/B ratio, − 6.23 to  − 10.90 for the 8:1 T/B ratio, − 4.23 to  − 9.39 for the 4:1 T/B ratio, respectively, Table 1). This indicated that the Gaussian filter had a large impact on quantifications. This study showed that there was no plateau of FWHM in terms of RCs, and RCs decreased dramatically along with the decrease of FWHM in all T/B ratios. Since there was no plateau for the Gaussian filter in terms of RCs, it was not used in the following analysis.

The AC + SC + RR combination presented a higher concentration concordance in spheres, and the AC + SC combination resulted in the lowest RCs (Fig. 5 and Table 2). Although RCs calculated with the AC + RR combination were higher than those of the AC + SC + RR and AC + SC combinations in all spheres, it resulted from the compensation of scattered photons with inaccurate energy and position information. For RCs of the largest 37 mm sphere calculated with the AC + RR combination in all T/B ratios, the plus tolerances were even greater than 20%. In contrast, these plus tolerances were only approximately 2.89–6.70% for the AC + SC + RR combination (Table 2). Since the scattered photons account for 30–40% of all photons acquired by the SPECT detector, the application of SC can reduce the errors of the calculated concentration to a great extent [61].

In principle, and among other factors, image quality in nuclear medicine is mainly affected by three factors: (1) spatial resolution (image sharpness) [62], (2) noise (variations in the image due to random effects such as quantum noise) [63], and (3) contrast (difference in image intensity between areas of the imaged object) [64]. The per cent contrast only measures the contrast aspect of the images but does not measure noise or spatial resolution, which altogether affects the image quality, as measured by lesion detectability. The resolution in this study was unchanged since the phantom did not have much anatomical variability. Thus, we added a noise evaluation (COV analysis) to the study in addition to the per cent contrast analysis to make the study more comprehensive and the results more indicative or potentially applicable to clinical situations. For image quality, the combination of AC + SC + RR had the best per cent contrasts in all T/B ratios (Table 3, all *P* values < 0.05). However, the COVs of the AC + SC + RR combination were higher than those of the AC + RR combination at all T/B ratios (Table 4, all *P* values < 0.05). The study of Knoll et al. also showed a similar result that the application of SC might increase the background variability [64]. This indicated that although AC + SC + RR combination resulted in the best quantitative performance, its image quality might be somewhat debatable. Since quantification was the main aim of this study, we selected AC + SC + RR combination as the optimal correction combination. Several reports also suggested the significance of AC, SC, and RR for SPECT quantification [29, 65–67]. Our study also showed that for all T/B ratios, COVs were relatively high. This was an inevitable compromise when quantification was the main aim of this study since a larger number of iterations could not only increase quantification accuracy but also increase background noise [47, 68].

For the 32:1 T/B ratio, RCs under the 15% energy window were higher than those under the 20% energy window, and there was a statistically significant difference (*P* value = 0.023). However, for lower T/B ratios (16:1, 8:1, and 4:1), there were no statistically significant differences (all *P* values > 0.05, Table 5). This suggested that although CZT SPECT/CT has a better image resolution due to the improved energy resolution of the new solid-state crystals [69], for RCs, the advantage of the 15% energy window might not be obvious enough for lower T/B ratios compared with that of the 20% energy window. Since a 15% energy window might improve the quantification accuracy for a higher T/B ratio (32:1), it was determined to be the optimal energy window in this step.

This study showed that the correlations between RCs and acquisition time were not obvious compared with those of other parameters. The regression coefficients between RCs and the acquisition time/frame were relatively small (− 0.01 to 0.07 for the 32:1 T/B ratio, − 0.04 to 0.28 for the 16:1 T/B ratio, − 0.31 to 0.12 for the 8:1 T/B ratio, and − 0.34 to 0.26 for the 4:1 T/B ratio, respectively, Table 1). RCs did not increase significantly with increasing acquisition time/frame (all *P* values > 0.05 when comparing 10 s with 120 s of acquisition time/frame). However, for the 4:1 T/B ratio, RCs of six spheres increased significantly within the first 40 s/frame of acquisition time (10 s vs. 120 s, *P* value = 0.015; 40 s vs. 120 s, *P* value = 0.060, Fig. 7). This suggested that for lower concentrations, the optimal acquisition time might be dependent on the activity concentration. Meanwhile, SD could be rapidly reduced within the first 40 s/frame of acquisition time (Fig. 8). These results suggested that acquisition time might only impose a strong impact on the quantification accuracy within this range. Therefore, 40 s/frame might be the optimal value in this step. In practice, 40 s/frame of acquisition time might be able to satisfy the quantification requirement.

The RCs of the 37 mm sphere reached 102.02–107.65% under the best acquisition and reconstruction parameters. However, these numbers dropped dramatically as the sphere volumes decreased (55.07–79.02% in the 10 mm sphere, Table 6). One possible reason is that the VOI counts decreased significantly with smaller objects due to the limitations brought on by the spatial resolution of SPECT [70].

There were four limitations to this study. First, the suggested acquisition and reconstruction parameters might only be applicable for quantitative purposes and for the CZT-SPECT equipment we investigated in this study. Second, due to the purpose of calculating mean uptake values with the lowest errors and determining the impacts of different acquisition and reconstruction parameters, VOIs were delineated using CT images as references. This procedure might be limited in clinical usage. Third, this study also showed that for all T/B ratios, COVs were relatively high. This was an inevitable compromise when quantification was the main aim of this study since a larger number of iterations could not only increase quantification accuracy but also increase background noise. Last, quantification measurements were only performed with a CZT-based camera system and not with a NaI (Tl)-based camera system, so a comparison between them was not evaluated.

## Conclusions

CZT-SPECT/CT of technetium showed a good quantification accuracy. The favourable acquisition parameters may be the 15% energy window and 40 s/frame. The favourable reconstruction parameters could be 35 iterations, 20 subsets, the AC + SC + RR correction combination, and no filter. Our results might have some merit for clinical quantification guidelines.

## Supplementary Information

**Additional file 1: Fig. S1** NEMA/IEC 2001 phantom. Black arrows, spheres with different diameters; White arrows, D-shaped cylinder

## Data Availability

The datasets used and/or analysed during the current study are available from the corresponding authors on reasonable request.

## Abbreviations

SPECT: Single-photon emission computed tomography
CZT: Cadmium–zinc–telluride
RC: Recovery coefficient
SD: Standard deviation
NEMA: National Electrical Manufacturers Association
FWHM: Full width at half maximum
AC: Attenuation correction
SC: Scatter correction
RR: Resolution recovery correction
PVC: Partial volume correction
NaI: Sodium iodide
PMT: Photomultiplier tube
PET: Positron emission computed tomography
VOI: Volume of interest
ROI: Region of interest
COV: Coefficient of variation
OSEM: Ordered subsets expectation maximization
