# Supplementary figures and images for: Impacts of acquisition and reconstruction parameters on the absolute technetium quantification of the cadmium–zinc–telluride-based SPECT/CT system: a phantom study
Source: EJNMMI Phys. 2021 Sep 26;8:66. doi: 10.1186/s40658-021-00412-4 (PMC8473509; doi:10.1186/s40658-021-00412-4)

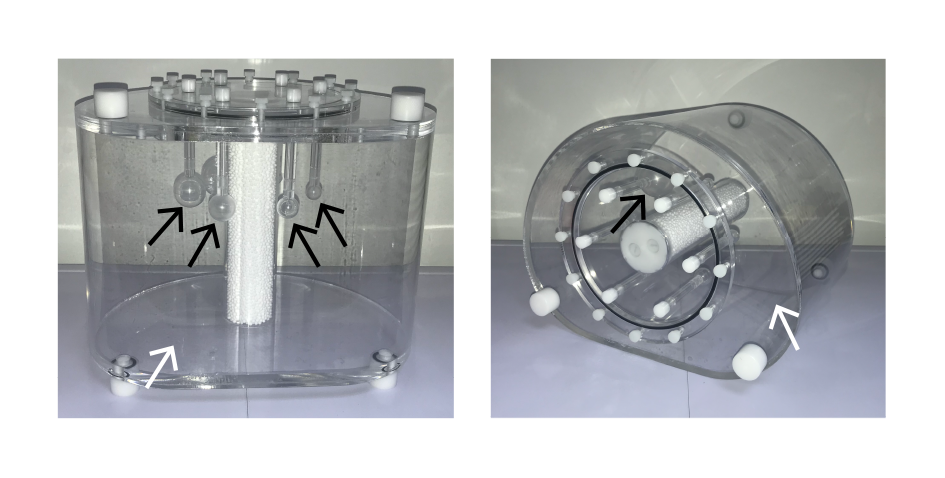

Supplement: Supplementary file 1 — Additional file 1: Fig. S1 NEMA/IEC 2001 phantom. Black arrows, spheres with different diameters; White arrows, D-shaped cylinder [file 40658_2021_412_MOESM1_ESM.tif]
